# IoT malware detection architecture using a novel channel boosted and squeezed CNN

**DOI:** 10.1038/s41598-022-18936-9

**Published:** 2022-09-15

**Authors:** Muhammad Asam, Saddam Hussain Khan, Altaf Akbar, Sameena Bibi, Tauseef Jamal, Asifullah Khan, Usman Ghafoor, Muhammad Raheel Bhutta

**Affiliations:** 1 Pattern Recognition Lab, Department of Computer and Information Sciences, Pakistan Institute of Engineering and Applied Sciences, Nilore, Islamabad, 45650 Pakistan; 2 PIEAS Artificial Intelligence Center (PAIC), Pakistan Institute of Engineering and Applied Sciences, Nilore, Islamabad, 45650 Pakistan; 3 Department of Computer Systems Engineering, University of Engineering and Applied Sciences, Swat, 19060 Pakistan; 4 Department of Economics, Management, Industrial Engineering and Tourism (DEGEIT), University of Aveiro, Aveiro, Portugal; 5 Department of Mathematics, Air University, Islamabad, 44000 Pakistan; 6 Department of Computer and Information Sciences, Pakistan Institute of Engineering and Applied Sciences, Nilore, Islamabad, 45650 Pakistan; 7 Center for Mathematical Sciences, Pakistan Institute of Engineering and Applied Sciences, Nilore, Islamabad, 45650 Pakistan; 8 Department of Mechanical Engineering, Institute of Space Technology, Islamabad, 44000 Pakistan; 9 School of Mechanical Engineering, Pusan National University, Busan, 46241 South Korea; 10 Department of Electrical and Computer Engineering, University of UTAH Asia Campus, Incheon, 21985 South Korea

**Keywords:** Computer science, Computational science

## Abstract

Interaction between devices, people, and the Internet has given birth to a new digital communication model, the internet of things (IoT). The integration of smart devices to constitute a network introduces many security challenges. These connected devices have created a security blind spot, where cybercriminals can easily launch attacks to compromise the devices using malware proliferation techniques. Therefore, malware detection is a lifeline for securing IoT devices against cyberattacks. This study addresses the challenge of malware detection in IoT devices by proposing a new CNN-based IoT malware detection architecture (iMDA). The proposed iMDA is modular in design that incorporates multiple feature learning schemes in blocks including (1) edge exploration and smoothing, (2) multi-path dilated convolutional operations, and (3) channel squeezing and boosting in CNN to learn a diverse set of features. The local structural variations within malware classes are learned by Edge and smoothing operations implemented in the split-transform-merge (STM) block. The multi-path dilated convolutional operation is used to recognize the global structure of malware patterns. At the same time, channel squeezing and merging helped to regulate complexity and get diverse feature maps. The performance of the proposed iMDA is evaluated on a benchmark IoT dataset and compared with several state-of-the CNN architectures. The proposed iMDA shows promising malware detection capacity by achieving accuracy: 97.93%, F1-Score: 0.9394, precision: 0.9864, MCC: 0. 8796, recall: 0.8873, AUC-PR: 0.9689 and AUC-ROC: 0.9938. The strong discrimination capacity suggests that iMDA may be extended for the android-based malware detection and IoT Elf files compositely in the future.

## Introduction

The concept of transforming real-world objects into virtual objects emerged as the Internet of Things (IoT). Under this concept, intelligent objects and devices can share data and resources according to the situation and environment^1^. This web of interconnected devices plays a vital role in our daily lives, ranging from health, smart homes, education, and, especially, industry. Masses are becoming familiar with the deployment of these devices in the field of agriculture, for soil condition monitoring^2^, healthcare and e-health applications^3–5^, and military domains^6^, as well. Deployments of these gadgets range from operational areas to critical infrastructure services. Industry 4.0 exploited this concept to build the link between the supply chain, industrial production, and end-users^7^. The IoT ecosystem used in industry, Industrial IoT (IIoT), undoubtedly contributes to the productivity and the quality of the industrial infrastructures.

**Figure 1:**
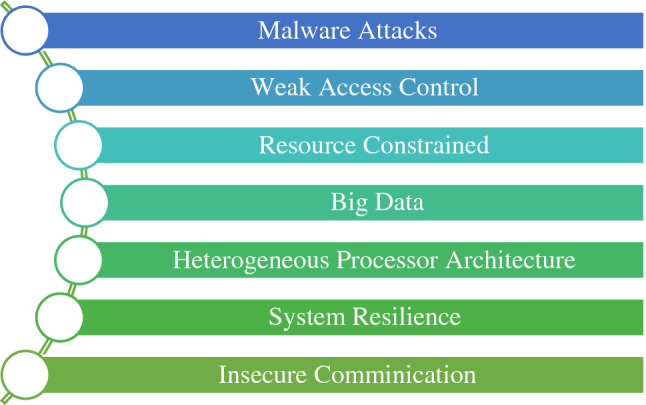
IoT security challenges.

IoTs lack secure design rules; hence, these have become an accessible playground for cybercriminals^8^. IoT devices are resource-constrained. These devices are usually installed with a default username password. Due to the embedded nature of the IoT devices, they are not patched regularly^9^. Network vulnerabilities for communicating with these devices can also be exploited easily at IoT touchpoints. The security protocol cannot be uniformly implemented on all the devices. The manufacturing of the devices does not conform to some consistent standards. These security challenges are depicted in Fig. 1.

There are many heterogenic device structures and network protocols. They also possess a unique characteristic of processor heterogeneity^10^. So, the IoT industry lags unified security protocols for design and implementation. These weaknesses of IoT design enlarge the attack surface area and lead to security breaches. Cybercriminals utilize these attack surfaces for their illegal actions and exploit the vulnerabilities. Major cyber security concerns are host and Network Intrusions, malware attacks, compromised nodes, botnets, rootkits, ransomware, and DDoS. Therefore, a robust mechanism for detecting such activities is needed to quickly detect and mitigate these digital security exploits.

Research on this security aspect of IoTs has attracted increased academic, industrial, and state-level attention. Several research efforts have discovered potential cyber threats and provided countermeasures against cyberattacks. Cyber security experts believe most cyber exploits are carried out through malware attacks. Many research studies in the literature have attempted this challenge of malware detection. Static, dynamic, hybrid, and image-based malware analysis comes under this challenge's broad categories^11^.

Machine learning techniques have been extensively used for malware detection as they are more robust and give promising performance^12–14^. Anti-malware tools have achieved improved performance with the help of machine learning tools. Several machine learning algorithms have been employed for mining the vulnerabilities in the IoT firmware and IoT applications that can infect and corrupt the edge devices and the whole network of the connected devices. Recent machine learning advancements have proved their capabilities in detecting and classifying IoT malware^15^. Research studies for anti-malware applications have increased the inclination towards machine learning tools and techniques. Computational power improvement has also enhanced the performance of machine learning strategies for malware detection and classification. Application of the machine learning needs the features of the IoT malware to make their verdict.

As the malware databases are increased, deep learning techniques suited more pertinent for the detection and analysis. Recent research has been molded towards applying neural networks in the field of malware analysis. Neural networks, especially deep convolution neural networks (CNNs), have proven their competencies for feature extraction and feature identification in IoT malware. Deep CNNs build the malware detection systems by defining the discriminative features in IoT malware. Deep CNNs show enhanced performance as these models learn the complicated features of the IoT malware at different abstraction levels. Features learned in the lower layers are enriched in the upper lawyers. These features are extracted from the visual images of the problem domain.

The IoT Malware dataset exploited in the current study has not been addressed previously to the best of our knowledge. This study utilized the image representation of IoTs malware and benign files. It is observed that deep CNN has shown promising performance for the visual challenges^16^. We have proposed applying deep learning techniques for the malware detection challenge. The main contributions in the current study are described below:

- A novel IoT Malware detection architecture (iMDA), using squeezing and boosting dilated CNN, is proposed for IoT Malware analysis using a new benchmark dataset.
- The proposed iMDA incorporates the edge and smoothing, multi-path dilated convolutional, channel squeezing, and boosting operations in CNN. Edge and smoothing operations are employed within split-transform-merge (STM) blocks to extract local structure and minor contrast variation in the malware images.
- STM blocks performed multi-path dilated convolutional operations, which helped to recognize the global structure of malware patterns. Additionally, channel squeezing and boosting are applied at different granular levels to get the reduced but prominent and diverse feature maps for capturing texture variations.
- The proposed iMDA has shown significant performance compared with existing CNNs via TL in terms of standard performance metrics using MCC, F1-Score, AUC, accuracy, precision, and recall.

The rest of the paper is structured as follows: the next section specifies related work in IoT malware analysis. “Methodology” section explains the proposed novel malware detection methodology. “Experimental setup” section describes the experimental setup.  “Results and discussion” section discusses the results of our work. The is described in “conclusion” section.

## Related work

IoT Malware analysis is carried out using static, dynamic, and hybrid analysis techniques. Nataraj et al.^17^ were the first to perform the malware analysis based on greyscale images in 2011. Malware visual images are created by transcribing the eight-bit code value of the executable files to the corresponding greyscale value. Image texture features are extracted from these images^18^. The idea of texture-based analysis for IoT malware is emerging in context with deep learning. Evanson et al.^19^ proposed an approach for malware analysis using texture images of malware files and machine learning in IoTPOT^20^ for Bashlite and Mirai. They came up with the Haralick image texture features from the grey-level co-occurrence matrix and used machine learning classifiers. Carrilo et al.^21^ explored the malware forensic and reverse engineering capabilities for malware characterization. They first used machine learning to detect Linux-based system malware of IoT. They also discovered new malware detection by using clustering techniques. They exploited the dataset provided by E. Cozzi et al.^22^. Ganesh et al.^23^ exploited machine learning capabilities to detect Mirai botnet attacks in IoTs. They applied ANN to evaluate their approach to the N-BaIoT dataset. Shudong Li et al.^24^ used ensemble learning for mining the malicious code in the cloud computing environment. Bendiab et al.^25^ applied deep learning for malware analysis traffic IoT. They applied ResNet50 for the experimental verification of their concept using a 1000 network (pcap) file.

Kyushu et al.^26^ proposed a lightweight approach for IoT malware detection. They targeted the DDoS malware for their study and extracted the malware images from malware binaries in IoTPOT^20^. Their experimental setup showed performance for detecting the DDoS malware and good-ware. Ren et al.^27^ gave an end-to-end malware detection mechanism for Android IoT devices. They collected 8000 benign and 8000 malicious APK files from the Google Play store and VirusShare, respectively. They used the significance of deep learning for the evaluation of their concept. Hussain et al.^28^ used application intent along with a supervised learning-based approach for the intelligent identification of Android-based malware. Naeem et al.^29^ detected the malware in Industrial IoT by proposing deep CNN-based traffic, behavior, and log databases analysis. They utilized the color images of the targeted malware for detection in the Leopard Mobile dataset.

Shafiq et al.^30^ used the Bot-IoT dataset for the correct malware feature selection and showed that their proposed method reached the accuracy of 965 for accurate detection of IoT malware over the network. They used the bijective soft set selection approach^31^ for the effective ML algorithm selection for the Bot-IoT network traffic dataset. They also used wrapper-based feature filtering and selection techniques^32^.

**Figure 2:**
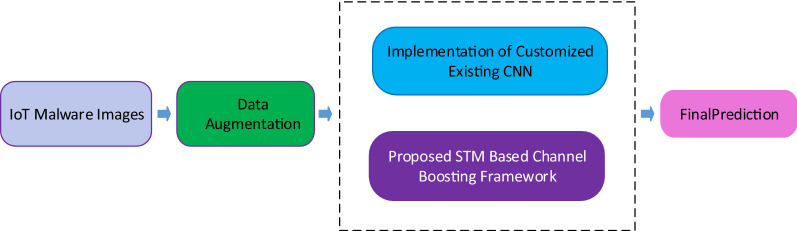
A brief overview of the proposed framework.

However, the evaluation of the reported work is presented in Accuracy and Precision. Practically, malware datasets are imbalanced. Therefore, other evaluation metrics must be considered. In this regard, our proposed research work exploited the benchmark Kaggle IoT dataset. Performance evaluation metrics F1-Score, MCC, AUC-PR, and AUC-ROC are also evaluated, along with Accuracy and Precision. The comprehensive workflow is presented in Fig. 2.

## Methodology

### Data augmentation

CNN models give better generalization upon large labeled data. Sometimes, the data points for the model training are not adequate. The data augmentation technique produces the artificial sample points by applying image transformation operations^33–35^. These operation includes rotation (0–360 degree), scaling (0.5–1), shearing (− 0.5, + 0.5) and reflection (in left and right direction). The augmentation process helped improve generalization and made the dataset more robust for detecting IoT malware.

### Proposed IoT malware detection architecture (iMDA)

This study proposes a novel image-based IoT malware detection architecture, iMDA. The suggested architecture discriminates the malware image sample from benign images. Spit-Transform-Merge (STM) is the main building block of this architecture. Three STM-based blocks concept is systematically implemented using region and edge detection operations.

**Figure 3:**
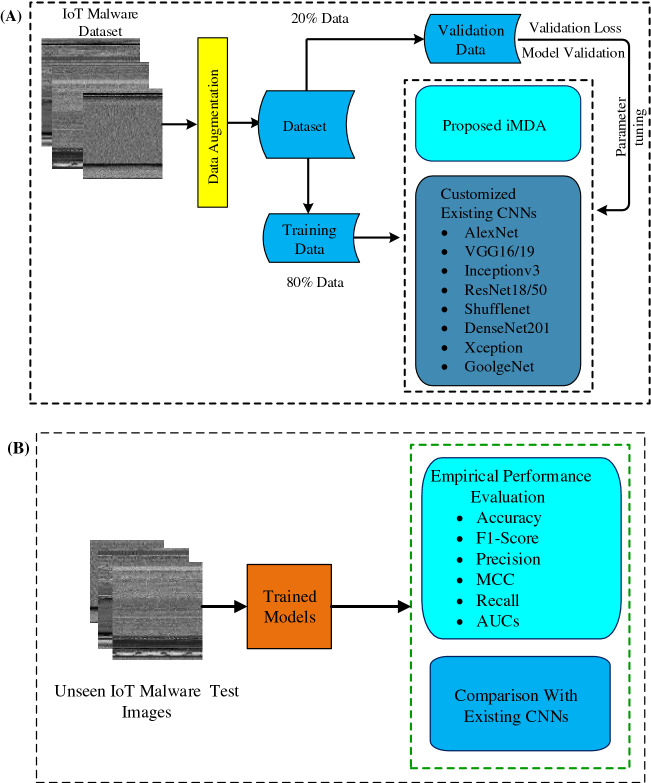
Detailed overview of training (**a**) and testing (**b**) of the proposed framework.

The concept of channel boosting is imparted for high precision, improving the detection rate. Implementation details of the proposed architecture are highlighted in Fig. 3A. The performance of the proposed architecture is compared with the existing CNN models using TL-based implementation, as shown in Fig. 3B.

### Proposed channel squeezing and boosting blocks

**Figure 4:**
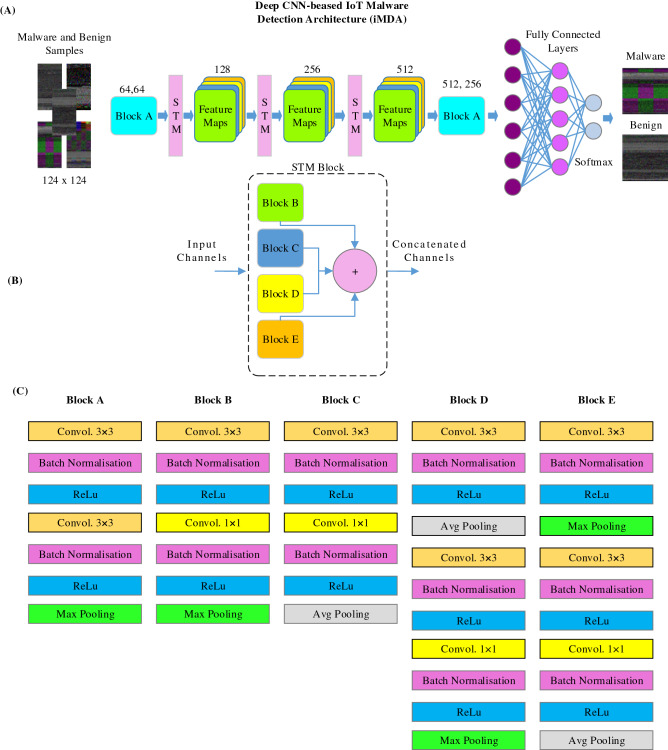
(**a**) The proposed IoT malware detection architecture (**b**) STM block (**c**) details of blocks used in the architecture.

Deep CNN models are powerful and robust for their texture feature mining abilities. These models use convolutional operations for exploiting structural information in the image data. These operations are used to extract the dataset's features according to the target domain. This innovative feature of the deep CNN is utilized in the current architecture for IoT malware detection. This architecture is tailored by proposing a concatenated STM-based channel boosting approach^36^, Fig. 4A.

The proposed STM block comprises a stack of four blocks, as shown in Fig. 4B. Details of the operations performed in each block are shown in Fig. 4C. Block B and Block C employ the same convolutions, batch normalization, and Relu operation with max and average pooling operation. Two convolution operations employed in each block are used to extract the feature information at the detailed and abstract levels, respectively. Block D and Block E employ the three-convolution operation. Two operations are used for the detailed features extraction, while one is used for the abstract level feature information extraction.

The STM block splits the input IoT malware image data into four branches to feed the four blocks of the STM. These blocks learn the region and edge-based informative features at a different level of abstraction from the input dataset. This learning helps to gather the highly discriminating features of the IoT malware at a high and detailed level. This info is imparted into different channels from each block. Information infused in other channels is concatenated at the exit of the STM block. This channel-boosted feature space is rich in diverse levels of textural feature information about the malware.

Equation (1) shows the convolution operation of filter f and input channel x of size p × q and A × B, respectively. The dimensions of the convolved output range from 1 to A − p + 1 and B—q + 1, respectively. ‘s’ denotes the dimension for average and max-pooling operations, shown in Eq. (2) and Eq. (3). In Eq. (4), CB, CC, CD, and CE show the channels extracted in the Block-B, -C, -D, and -E, respectively. The ‘merge’ function is used for concatenating these extracted channels. u*d* shows the number of neurons in Eq. (5).

1 $$x_{a,b} = \mathop \sum \limits_{i = 1}^{p} \mathop \sum \limits_{j = 1}^{q} x_{a + i - 1,b + j - 1} * f_{i,j}$$

2 $$\left( {xavg} \right)_{a,b} = \frac{1}{{s^{2} }}\mathop \sum \limits_{i = 1}^{s} \mathop \sum \limits_{j = 1}^{s} x_{a + i - 1,b + j - 1}$$

3 $$\left( {x\max } \right)_{a,b} = \max_{i = 1, \ldots s,j = 1, \ldots s} x_{a + i - 1, b + j - 1}$$

4 $$C_{Boost} = merge(C_{B} \left| {\left| {C_{C} } \right|} \right|C_{D} ||C_{E} )$$

5 $$x = \mathop \sum \limits_{d = 1}^{D} \mathop \sum \limits_{c = 1}^{C} u_{d} x_{c}$$

### Implementation of customized existing CNNs

CNN architectures AlexNet, VGG16, inceptionv3, VGG19, Resnet50, Shufflenet, DenseNet201, Xception, and GoolgeNet are selected for a fair comparison with the proposed architecture. To achieve substantial performance, these models are initially trained on the ImageNet. These trained models are fine-tuned according to the target IoT malware dataset. Then these models are trained and tested using the target dataset using an 80-20 train-test split.

## Experimental setup

### Dataset

**Figure 5:**
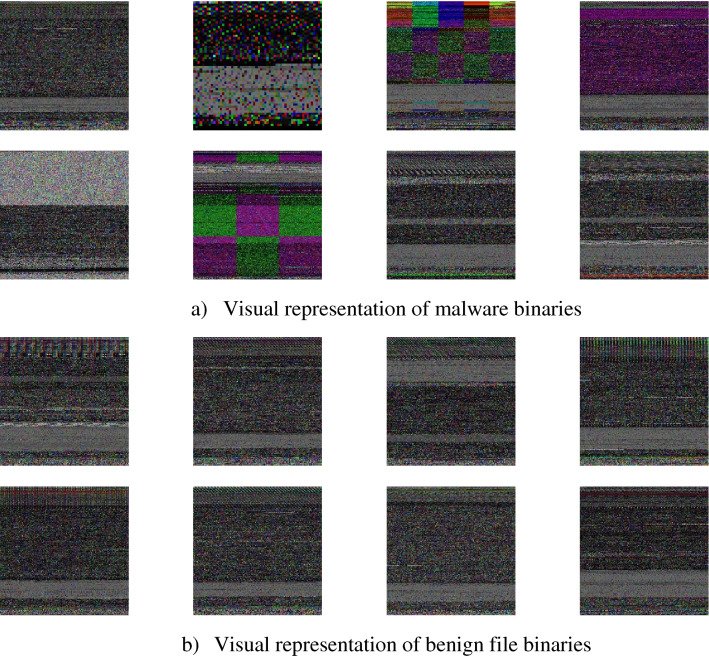
Image visualization of (**a**) malware and (**b**) benign files.

Linux operating system (OS) is becoming the dominant for IoT devices^37^. Hence, this operating system has become a prospecting target for the malware developer community. Linux uses ELF file format for the deployment of applications or firmware. ELF files are cross plate form in nature and come in two binary formats, packed and unpacked binaries^38^. The IOT_Malware dataset used in this study is the image representation of unpacked ELF binary files for malware and benign applications^39^. This dataset is a standard Kaggle benchmark dataset for IoT malware detection challenges. There are 14,733 greyscale images of malware application ELF binaries and 2486 greyscale images of legitimate application ELF binaries. Visualization of benign and malicious files is shown in Fig. 5.

### Implementation details

The implementation of the proposed iMDA is simulated using MATLAB-2021a on Nvidia® GTX 1060-T, GPU-enabled Dell Core I i5-7500. It took ~ 1–2 h to train a model on the said settings. One epoch took 7–10 min on Nvidia-Tesla K-80, while a single IoT malware image took approximately 2 s for detection.

### Performance evaluation metrics

In the current study, we have employed performance evaluation metrics Accuracy, Precision, Recall, F1-Score, and MCC, as shown in Eqs. (6–10). The details of these performance metrics are described in Table 1. AUC-PR and AUC-ROC is also formulated for the proposed model. True Positive (TP), False Positive (FP), True Negative (TN), and False Negative (TN) are also calculated for the performance comparison.

6 $${\text{Acc}} = \frac{{{\text{Predicted}}\;{\text{malware}}\;{\text{samples }} + {\text{predicted}}\;{\text{benign}}\;{\text{samples}}}}{{{\text{Total}}\;{\text{samples}}}} \times 100$$

7 $${\text{MCC}} = \frac{{\left( {{\text{TP}}*{\text{TN}}} \right) - \left( {{\text{FP}}*{\text{FN}}} \right)}}{{\sqrt {\left( {{\text{TP}} + {\text{FP}}} \right)*\left( {{\text{FP}} + {\text{FN}}} \right)*\left( {{\text{TN}} + {\text{FP}}} \right)*\left( {{\text{TN}} + {\text{FN}}} \right)} }}$$

8 $${\text{P}} = \frac{{{\text{Predicted}}\;{\text{malware}}\;{\text{samples}}}}{{{\text{Predicted}}\;{\text{malware}}\;{\text{samples}} + {\text{ Incorrectly}}\;{\text{predicted}}\;{\text{Malware}}\;{\text{samples}}}} \times 100$$

9 $${\text{R}} = \frac{{{\text{Predicted}}\;{\text{malware}}\;{\text{samples}}}}{{{\text{Total}}\;{\text{malware}}\;{\text{samples}}}} \times 100$$

10 $${\text{F}}1 - {\text{Score}} = 2 \times \frac{{{\text{P}} \times {\text{R}}}}{{{\text{P}} + {\text{R}}}}$$

**Table 1:** Details of performance metrics.

Metric symbol	Description
Acc	Shows Accuracy as % of the total number of Malware detection
R	Shows Recall, which is the proportion of correctly identified malware samples and benign samples
P	Shows Precision, a ratio of correctly detected malware samples to the total malware sample
F1-Score	F1-Score is the harmonic mean of P and R
AUC-PR	Quantifies the area under Precision and Recall Curve
AUC-ROC	Quantifies the area under Receiver Operating Characteristic curve
MCC	Mathews Correlation Coefficient
TP	Correctly Identified Malware Files
TN	Correctly Identified Benign Files
FP	Incorrectly Identified Malware Files
FN	Incorrectly Identified Benign Files

Several statistical measures are used as performance metrics for binary classification using four quadrants confusion matrix, i.e., TP, TN, FP, and FN. These metrics are selected according to the problem under investigation. There is no agreed-upon performance metrics for two or multi-class problem. The severity of the problem gives direction toward the selection of performance metrics. For an imbalanced dataset, some performance metrics show over-optimistic results. The Matthews correlation coefficient (MCC) is considered an attested statistical measure. It gives a high score for prediction only if all four quadrants are proportionally high for both positive and negative classes^40^.

## Results and discussion

### Performance analysis of the proposed iMDA

The performance of the proposed iMDA is assessed on a standard IoT Malware dataset. F1-Score and MCC are considered standards for performance evaluation for an imbalanced dataset. F1-Score and MCC are used for assigning weightage to both the precision and sensitivity. The proposed architecture converged smoothly and reached the optimal value quickly, as shown in the training plots of the model. Misclassification occurred due to the intrinsic code similarity between the malicious and benign files. This similarity refers to the identical attack pattern in the malware images. This phenomenon occurred substantially with the implementation of other CNN models for malware detection. The iMDA is carried out using data augmentation techniques that improve the generalization and robustness of the trained model during testing.

### Performance comparison with existing CNNs

The performance of the IoT malware detection architecture, iMDA, is also compared with existing models, AlexNet, VGG16, inceptionv3, VGG19, Resnet50, Shufflenet, DenseNet201, Xception, and GoolgeNet. Improved performance is shown in Table 2.

**Table 2:** Comparison of proposed framework with the existing CNN models.

Models	Accuracy %	F1-score	Precision	MCC	Recall	AUC-PR	AUC-ROC
AlexNet	92.86	0.6807	0.9960	0.5874	0.5171	0.9041	0.9685
VGG16	94.72	0.9146	0.9552	0.839	0.8772	0.9321	0.9816
Inceptionv3	94.89	0.8055	0.9920	0.7091	0.6780	0.8972	0.9860
VGG19	95.38	0.8353	0.9902	0.7429	0.7223	0.9088	0.9739
Resnet50	95.62	0.8282	0.9971	0.7379	0.7082	0.9432	0.9848
Shufflenet	95.93	0.8491	0.9949	0.7621	0.7404	0.9541	0.9901
DenseNet201	96.17	0.8685	0.9917	0.7856	0.7726	0.9471	0.9884
Xception	96.57	0.9342	0.9737	0.8651	0.9074	0.9527	0.9882
GoolgeNet	96.72	0.8934	0.9917	0.8195	0.8128	0.9469	0.9881
Proposed iMDA	97.93	0.9394	0.9864	0.8796	0.8873	0.9731	0.9938

CNN models seek to find the identifiable textures and patterns in the image dataset. Our proposed malware detection architecture, iMDA, better explored textural variation in the malware images by systematically using region and boundary information through the Avg and Max-pooling operations. Channel split-transform-merge technique helped to extract the features at different granularity. Incorporating the concepts mentioned earlier in CNN improved the performance of the proposed architecture over the existing models. This study reported the significance of performance using deep learning architecture and quantified it using MCC, F1-Score, AUC-ROC, Accuracy, Precision, and Recall.

### Detection capability of the proposed iMDA

**Figure 6:**
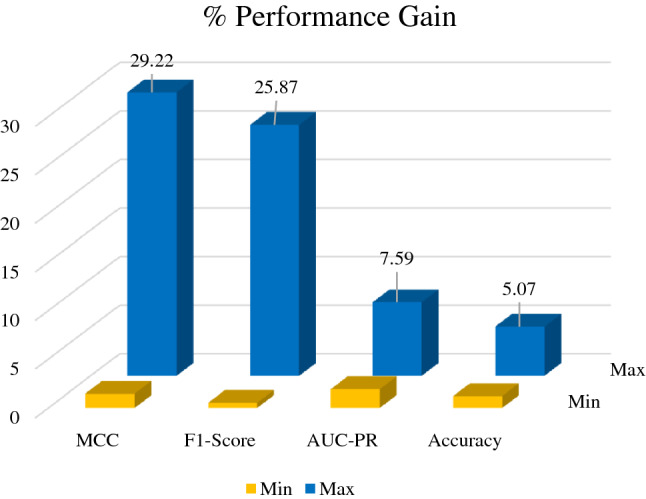
Minimum and maximum performance gain of proposed framework.

**Figure 7:**
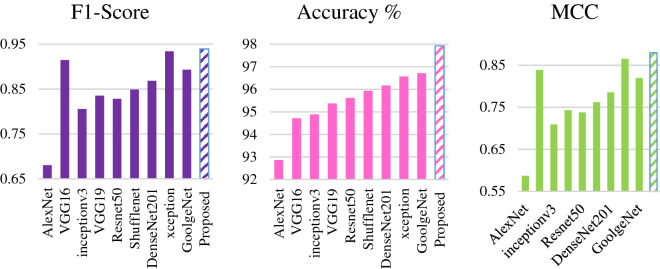
F1-score, accuracy, and MCC comparison.

The effectiveness of a malware detection framework is mainly assessed through precision rate and detection rate. Accurately detecting infused malware in a system is the first parameter to secure and control the spread. False alarms may be increased if only the precision of the proposed detection technique is improved. Decreasing the false alarm may degrade the detection rate. Keeping in mind this intuition, the proposed model leveraged the difference by comparing F1-Score, the harmonic mean of both parameters. Minimum and maximum performance gains against the existing CNN models are shown in Fig. 6. Results of the proposed iMDA are summarized in Table 3. A comparison of detection performance of our proposed model using F1-Score, Accuracy, and MCC with the existing model is shown in Fig. 7. In contrast, customized existing CNNs are compared and found that few models showed considerably good precision with poor recall.

**Table 3:** Performance of the proposed model.

Performance metric	Proposed iMDA
Accuracy %	97.93
F1-Score	0.9394
Precision	0.9864
MCC	0.8796
Recall	0.8873
AUC-PR	0.9689
AUC-ROC	0.9938

### Feature space-based analsis of the proposed iMDA

**Figure 8:**
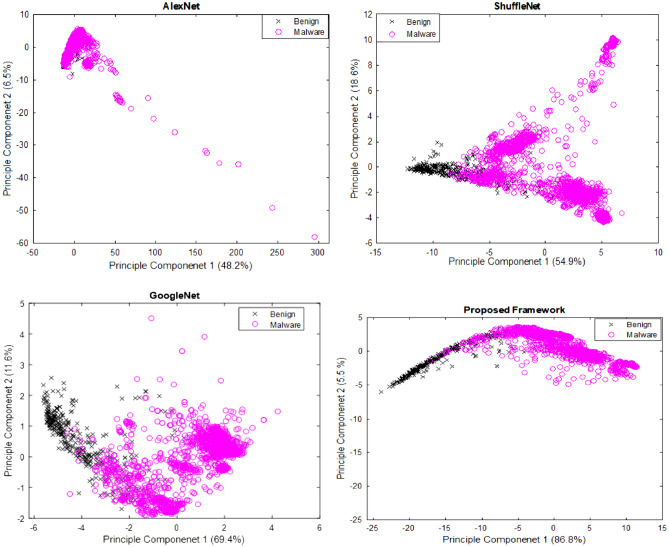
Feature space-based performance comparisons.

The decision-making of the proposed architecture is better analyzed with the help of feature space visualization. Better discrimination factor of the model is associated with the prominent visual features. This distinction helps to improve the learning and lower the variance of the model. The feature space visualization for the principal components of our proposed iMDA is shown in Fig. 8. Channel squeezing and channel boosting used in STM blocks helped to capture the discriminative features of the IoT malware images at a multi-level. Additionally, STM extended the reduced prominent feature with the help of channel concatenation. The feature space visualization for the proposed iMDA showed an improvement in identifying the distinct and diverse features, hence improving the detection of the IoT malware files.

### Auc-roc and auc-pr based analysis

**Figure 9:**
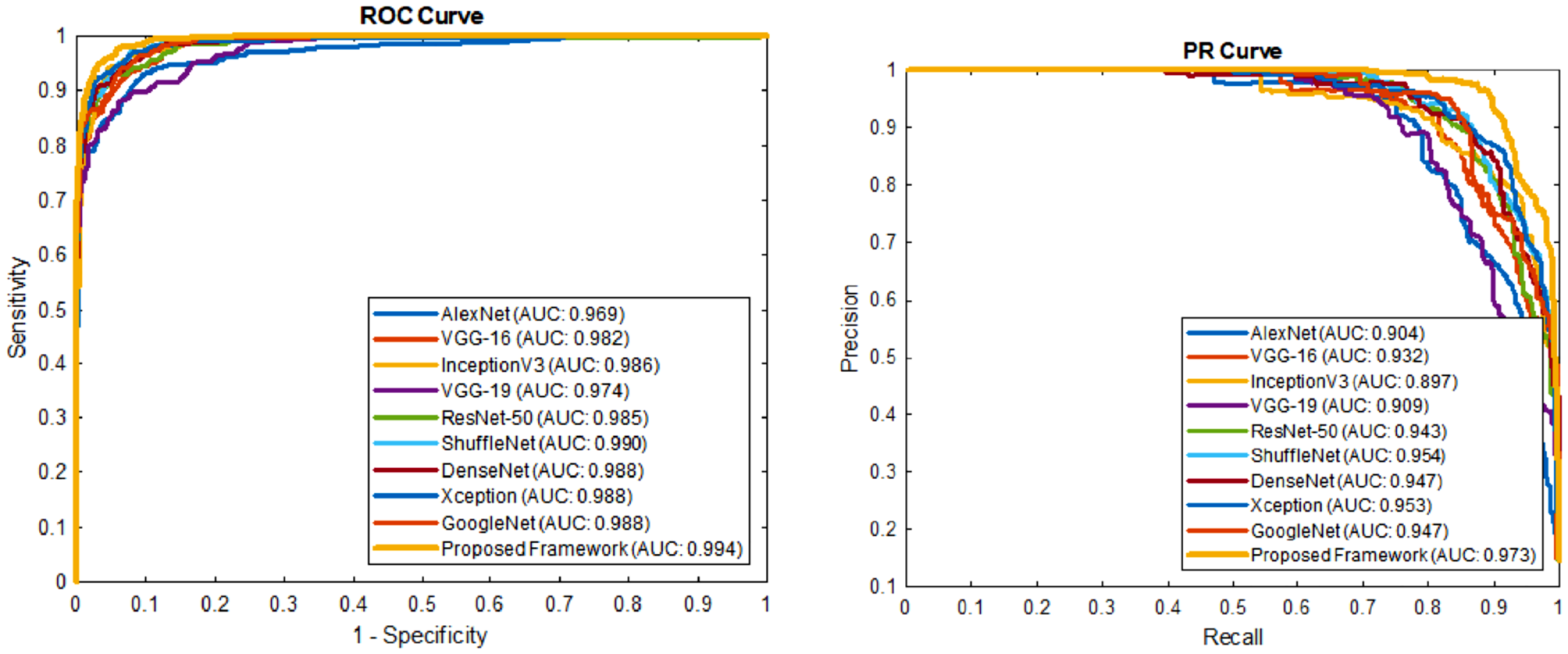
Detection rate analysis of the proposed iMDA in comparison with existing CNN.

The optimal performance of the model is also best understood by the ROC and PR plots, Fig. 9. These plots show the bifurcation capability of the models at an optimal threshold value. Our proposed iMDA showed high sensitivity along with a decreased false positive rate.

## Conclusion

Analysis of malware in IoT is an early line of defense in securing this world of connected devices from cyberattacks. Malware analysis help to identify and designate the malicious code segments hidden in the legitimate files. This malicious code snippet is present according to the malware signature or obfuscated otherwise. The obfuscation techniques hide the malicious code lines from pattern/signature matching. These lines may be distributed or intermixed with the legitimate line over the complete file. The IoT-specific malicious patterns are detected in this study by developing iMDA, new CNN architecture: iMDA based on the ideas of dilated convolutional operations, channel squeezing, and boosting. The proposed architecture discriminates the malware from benign based on textural, contrast, and pattern variations. The proposed iMDA outperformed existing CNN and achieved the best result for Accuracy (97.33%), MCC (0.8796), F1-Score (93.94), AUC-ROC (0.9938), and AUC-PR (0.9689). In the future, the proposed iMDA may be extended for the android-based malware detection and IoT Elf files compositely.

## Data Availability

The datasets used and/or analyzed during the current study available from the corresponding author on reasonable request.

## References

[1] Madakam S, Ramaswamy R, Tripathi S (2015). Internet of things (IoT): A literature review. J. Comput. Commun..

[2] Vuran MC, Salam A, Wong R, Irmak S (2018). Internet of underground things in precision agriculture: Architecture and technology aspects. Ad Hoc Netw..

[3] Zafar MM (2022). Detection of tumour infiltrating lymphocytes in CD3 and CD8 stained histopathological images using a two-phase deep CNN. Photodiagnosis Photodyn. Ther..

[4] Islam SMR, Kwak D, Kabir MH, Hossain M, Kwak KS (2015). The internet of things for health care: A comprehensive survey. IEEE Access.

[5] Zahoor, M. M., Qureshi, S. A., Khan, S. H. & Khan, A. A New Deep Hybrid Boosted and Ensemble Learning-based Brain Tumor Analysis using MRI (2022). https://arxiv.org/abs/2201.05373 10.3390/s22072726 PMC9002515 35408340

[6] Iyer B, Patil N (2018). IoT enabled tracking and monitoring sensor for military applications. Int. J. Syst. Assur. Eng. Manag..

[7] Mikhalevich, I. F. & Trapeznikov, V. A. Critical infrastructure security: alignment of views. In *2019 Systems of Signals Generating Processing in the Field of on Board Communication. SOSG 2019* 1–5 (2019). 10.1109/SOSG.2019.8706821

[8] Shao Z, Yuan S, Wang Y (2021). Adaptive online learning for IoT botnet detection. Inf. Sci. (Ny).

[9] Ngo QD, Nguyen HT, Le VH, Nguyen DH (2020). A survey of IoT malware and detection methods based on static features. ICT Express.

[10] Vignau B, Khoury R, Hallé S, Hamou-Lhadj A (2021). The evolution of IoT malwares, from 2008 to 2019: Survey, taxonomy, process simulator and perspectives. J. Syst. Archit..

[11] Asam M (2021). Detection of exceptional malware variants using deep boosted feature spaces and machine learning. Appl. Sci..

[12] Or-Meir O, Cohen A, Elovici Y, Rokach L, Nissim N (2021). Pay attention: Improving classification of PE malware using attention mechanisms based on system call analysis. Proc. Int. Jt. Conf. Neural Netw..

[13] Asam, M., Hussain Khan, S., Jamal, T., Zahoora, U. & Khan, A. Malware Classification Using Deep Boosted Learning.

[14] Rafique, M. F., Ali, M., Qureshi, A. S., Khan, A. & Mirza, A. M. Malware Classification using Deep Learning based Feature Extraction and Wrapper based Feature Selection Technique, Oct. 2019, Accessed: Jun. 20, 2021. [Online]. Available: http://arxiv.org/abs/1910.10958

[15] Li S, Zhang Q, Wu X, Han W, Tian Z (2021). Attribution classification method of APT malware in IoT using machine learning techniques. Secur. Commun. Netw..

[16] Khan A, Sohail A, Zahoora U, Qureshi AS (2020). A survey of the recent architectures of deep convolutional neural networks. Artif. Intell. Rev..

[17] Nataraj L, Karthikeyan S, Jacob G, Manjunath BS (2011). Malware images: Visualization and automatic classification. ACM Int. Conf. Proc. Ser..

[18] Ma, Y., Liu, S., Jiang, J., Chen, G. & Li, K. *A Comprehensive Study on Learning-Based PE Malware Family Classification Methods*, vol. 1, 1. Association for Computing Machinery (2021).

[19] Karanja EM, Masupe S, Jeffrey MG (2020). Analysis of internet of things malware using image texture features and machine learning techniques. Internet Things (Netherlands).

[20] Pa, Y. M., Suzuki, S., Yoshioka, K., Matsumoto, T., Kasama, T. & Rossow, C. IoTPOT: Analysing the rise of IoT compromises. In *9th USENIX Work. Offensive Technology WOOT 2015* (2015).

[21] Carrillo-Mondéjar J, Martínez JL, Suarez-Tangil G (2020). Characterizing Linux-based malware: Findings and recent trends. Futur. Gen. Comput. Syst..

[22] Cozzi, E., Graziano, M., Fratantonio, Y. & Balzarotti, D. Understanding Linux malware. In *Proceedings of IEEE Symposium Secure Privacy*, vol. 2018-May, 161–175 (2018). 10.1109/SP.2018.00054

[23] Palla, T. G. & Tayeb S. Intelligent Mirai Malware Detection in IoT Devices. In *2021 IEEE World AI IoT Congress AIIoT 2021*, 420–426 (2021). 10.1109/AIIoT52608.2021.9454215

[24] Li S, Li Y, Han W, Du X, Guizani M, Tian Z (2021). Malicious mining code detection based on ensemble learning in cloud computing environment. Simul. Model. Pract. Theory.

[25] Bendiab, G., Shiaeles, S., Alruban, A. & Kolokotronis, N. IoT malware network traffic classification using visual representation and deep learning. In *Proceedings of 2020 IEEE Conference on Network Softwarization Bridge Gap Between AI Network Softwarization, NetSoft 2020* 444–449 (2020). 10.1109/NetSoft48620.2020.9165381.

[26] Su J, Danilo Vasconcellos V, Prasad S, Daniele S, Feng Y, Sakurai K (2018). Lightweight classification of IoT malware based on image recognition. Proc. Int. Comput. Softw. Appl. Conf..

[27] Ren Z, Wu H, Ning Q, Hussain I, Chen B (2020). End-to-end malware detection for android IoT devices using deep learning. Ad Hoc Netw..

[28] Hussain SJ, Ahmed U, Liaquat H, Mir S, Jhanjhi NZ, Humayun M (2019). IMIAD: Intelligent malware identification for android platform. Int. Conf. Comput. Inf. Sci. ICCIS.

[29] Naeem H (2020). Malware detection in industrial internet of things based on hybrid image visualization and deep learning model. Ad Hoc Netw..

[30] Shafiq M, Tian Z, Bashir AK, Du X, Guizani M (2021). CorrAUC: A malicious Bot-IoT traffic detection method in IoT network using machine-learning techniques. IEEE Internet Things J..

[31] Shafiq M, Tian Z, Sun Y, Du X, Guizani M (2020). Selection of effective machine learning algorithm and Bot-IoT attacks traffic identification for internet of things in smart city. Futur. Gen. Comput. Syst..

[32] Shafiq M, Tian Z, Bashir AK, Du X, Guizani M (2020). IoT malicious traffic identification using wrapper-based feature selection mechanisms. Comput. Secur..

[33] Shorten C, Khoshgoftaar TM (2019). A survey on image data augmentation for deep learning. J. Big Data.

[34] Wang, J. & Perez, L. The Effectiveness of Data Augmentation in Image Classification using Deep Learning (2017).

[35] Hussain Khan, S., Khan, A., Soo Lee, Y., Hassan, M. & Kyo Jeong, W. Segmentation of Shoulder Muscle MRI Using a New Region and Edge Based Deep Auto-Encoder.

[36] Khan SH, Sohail A, Khan A, Lee Y-S (2022). COVID-19 detection in chest X-ray images using a new channel boosted CNN. Diagnostics.

[37] E. Foundation. Iot-Comm-Adoption-Survey-2019 (2020).

[38] Wan, T. L. *et al.*, IoT-malware detection based on byte sequences of executable files. In *2020 15th Asia Joint Conference on Information Security (AsiaJCIS 2020)* 143–150 (2020). 10.1109/AsiaJCIS50894.2020.00033

[39] Elmasry, A. IOT_Malware, https://www.kaggle.com/anaselmasry/iot-malware (accessed Aug. 08, 2021).

[40] Chicco, D. & Jurman, G. The advantages of the Matthews correlation coefficient (MCC) over F1 score and accuracy in binary classification evaluation 1–13 (2020). 10.1186/s12864-019-6413-7 PMC6941312 31898477

